# Low-Temperature Stress Signatures in Maize During Emergence: Linking Osmoprotectants, Antioxidative Enzymes, and Photosynthetic Performance

**DOI:** 10.3390/biology15181634

**Published:** 2026-09-16

**Authors:** Manja Božić, Dragana Ignjatović Micić, Marija Kostadinović, Mirjana Milovanović, Nikola Grčić, Petar Čanak, Ana Nikolić

**Affiliations:** 1 Laboratory for Molecular Genetics and Physiology, Maize Research Institute “Zemun Polje”, 11185 Belgrade, Serbia; idragana@mrizp.rs (D.I.M.); kmarija@mrizp.rs (M.K.); mmilovanovic@mrizp.rs (M.M.); anikolic@mrizp.rs (A.N.); 2 Maize Breeding Group, Maize Research Institute “Zemun Polje”, 11185 Belgrade, Serbia; ngrcic@mrizp.rs; 3 Seed Production Sector, Maize Research Institute “Zemun Polje”, 11185 Belgrade, Serbia; pcanak@mrizp.rs

**Keywords:** maize, emergence stage, low-temperature stress response, osmoprotection, antioxidant response, photosynthesis

## Abstract

Early sowing is one of the most promising strategies for mitigating the adverse effects of climate change on maize production in temperate regions. A rapid and strong initial response is critical for seedling survival and can indicate early-sowing tolerance. Consequently, a better understanding of the early mechanisms underlying the establishment of low-temperature (LT) tolerance during early development in maize, including the emergence stage, becomes essential. However, research data on the LT response during emergence are limited. In this study, 5-day-old seedlings of two maize lines with contrasting tolerance to LT (L_S_ and L_T_) were subjected to low-temperature treatment (10/8 °C) for 24 h, after which metabolites important for LT response were profiled (phenolic compounds, sugars, and sugar alcohols). Activities of several antioxidative enzymes, as well as photosynthetic efficiency, were also examined. Trehalose and inositol accumulation as well as increased peroxidase and superoxide dismutase activities were common to both genotypes. A significant decrease in photosynthetic efficiency, as well as flavonoid and glucose accumulation, was detected only in L_S_, while sorbitol and erythritol accumulated only in L_T_. The findings show that both genotypes share core LT responses, while genotype-specific metabolic and photosynthetic adjustments distinguish LT tolerance during the emergence stage.

## Simple Summary

Knowing how young maize plants respond to low temperatures during early developmental stages, such as emergence, is extremely important in aiming to support earlier sowing as a way to mitigate climate change impacts on maize production. Still, information on the low-temperature response during emergence is limited compared to later developmental stages. In the present study, the response of sensitive and tolerant seedlings was inspected, showing distinct differences. Seedlings susceptible to low temperatures showed reduced photosynthesis and accumulation of compounds that signal inhibited growth, while the same was not found for the tolerant ones. This knowledge can be used in designing maize breeding programs that are important for early sowing.

## 1. Introduction

Climate change caused by increasing greenhouse gas emissions exacerbates abiotic stresses such as drought, high and low temperatures, flooding, and salinity. Consequently, the adverse effects of climate change are evident in agriculture, categorizing it as one of the most susceptible sectors, leading to reduced yield and food insecurity. Addressing these negative impacts on plants necessitates the implementation of various physical, chemical, biological, and agronomic strategies, as well as their combinations, to enhance crop resilience [1].

Maize ranks among the top three staple cereals globally, alongside wheat and rice, and is expected to become the most widely cultivated and traded crop in the near future [2]. As with other crops, the effects of abiotic stress factors caused by climate change are among the most challenging factors for maize production. Drought and heat stress are the most influential and detrimental abiotic stresses when it comes to maize production worldwide. Heat stress during the late vegetative phases reduces maize yield by as much as 30%. Conversely, during the flowering and lag phases (the initial grain-filling stage), yield reductions can be as high as 50% [3]. Finally, the combination of heat and drought stress can result in significantly greater yield losses. Early maize sowing could improve yields by avoiding periods of extreme heat and drought in summer, but the risks of negative impacts from low-temperature (LT) exposure during early developmental stages, such as emergence, are significantly elevated. The emergence stage is considered extremely thermo-sensitive, and LT stress can lead to delayed germination, reduced photosynthetic capacity, and significant biomass loss, ultimately compromising grain yield [4,5]. Additionally, LT stress at that stage does affect maize yield [6,7]. Specifically, as early-sown maize encounters low temperatures when the seedlings are very young and vulnerable, the speed and strength of the initial response are of great importance for survival and can be a useful indicator of early-sowing tolerance. However, most research studies on the LT response in maize during early development have focused on germination or later stages, such as V3 [8,9,10,11], while not many were aimed at the emergence stage [12,13]. As each growth stage of maize is genetically independently controlled, LT tolerance might also be regulated independently in the different growth stages [14], emphasizing the importance of fully grasping all aspects of LT response at this stage, particularly the initial response, including osmoprotectant accumulation and antioxidative enzyme activities.

Exposure to low temperatures initially causes various disruptions in the regular metabolic processes of plants. Low temperatures first exert their effect on cell membranes, increasing their permeability and reducing fluidity, which further leads to disturbances in membrane transport, photosynthesis and osmotic potential. The photosynthetic apparatus is highly susceptible to low temperatures, especially photosystem II (PSII). Certain chlorophyll fluorescence parameters, such as the maximum quatum yield (F_v_/F_m_) and the electron transfer rate (ETR), can serve as non-invasive indicators of LT-induced damage to the photosynthetic apparatus [15]. Low temperatures also cause the formation of reactive oxygen species (ROS), which can cause oxidative damage to lipids, carbohydrates, proteins, and nucleic acids, as well as photoinhibition, pigment degradation and loss of enzyme activity. The metabolic and physiological disruptions further lead to tissue damage and impairments in the development of plant organs, which eventually cause growth inhibition, reductions in plant height and root length, leaf yellowing, wilting, necrosis, and finally the death of seedlings [14].

Still, when plants are exposed to low temperatures, they undergo a series of physiological and biochemical changes that form the plants’ response to the stress factor and maintain cellular homeostasis. These physiological and biochemical changes can play a role in the early, rapid response necessary to ensure the plants’ immediate survival, as well as in the delayed long-term acclimation processes that ensure long-term functioning under stress conditions [16,17]. The focus of the present study was the early, acute response, which included antioxidative mechanisms and accumulation of protective solutes, such as osmoprotectants. To counteract the overproduction of ROS, plants have evolved a sophisticated antioxidative defense system, that includes enzymatic antioxidants such as superoxide dismutase (SOD), peroxidase (POD), catalase (CAT), glutathione peroxidase (GPX), and ascorbate peroxidase (APX), as well as non-enzymatic antioxidants like phenolic compounds and flavonoids [18]. Phenolic acids and flavonoids are known to scavenge free radicals and stabilize membranes, but their specific accumulation patterns under low-temperature stress often vary significantly between tolerant and susceptible genotypes [19]. In addition to the antioxidative response, soluble sugars (sucrose, raffinose, trehalose, etc.) and sugar alcohols (sorbitol, inositol, etc.) play a vital role in cryoprotection, as they act as osmoprotectants or compatible solutes. Osmoprotectants have a role in stabilizing cellular membranes, maintaining turgor pressure through osmotic adjustment, and protecting the photosynthetic apparatus from stress-induced photoinhibition [14]. Therefore, low-temperature stress induces excessive ROS production, triggering antioxidant defenses and the accumulation of protective osmolytes to stabilize cellular structures. These response mechanisms help mitigate photoinhibition and maintain photosynthesis under low temperatures, ultimately determining low-temperature tolerance.

The aim of the present study was to investigate the contrasting early responses of two maize inbred lines—one tolerant and one susceptible to low-temperature stress—at the critical five-day-old seedling stage. The focus was to determine whether the initial and rapid changes in a range of metabolites and enzymes known to play significant roles in the abiotic stress response in maize during different developmental stages also responded to low-temperature treatment after 24 h of exposure during the emergence stage. This included analyzing the contents of phenolic compounds, soluble sugars, sugar alcohols, and antioxidative enzyme activities, as antioxidant defense and osmolyte accumulation are important aspects of the short-term stress response in plants [20,21]. Additionally, photosynthetic efficiency and recovery were assessed to estimate the metabolic damage caused by low-temperature stress. The correlations among these components were also evaluated to uncover their potential roles in maize low-temperature tolerance during this less explored developmental stage. This research provides insights into the biochemical signatures of tolerance during the emergence stage that could be utilized in future focused studies, enabling the development of more resilient maize genotypes in the face of climate change.

## 2. Materials and Methods

### 2.1. Plant Material and Low-Temperature Treatment

Maize seedlings belonging to two maize inbred lines (genotypes) with contrasting tolerance to low temperatures were subjected to this stress factor during the emergence stage, specifically at the five-day-old seedling stage. Both lines are temperate maize and parental components of commercial hybrids, developed at the Maize Research Institute Zemun Polje (Belgrade, Serbia). The tolerant inbred line (L_T_) was a semi-dent from the BSSS heterotic group (FAO 650), while the susceptible inbred line (L_S_) was a dent and belonged to the Lancaster heterotic group (FAO 350). The contrasting LT tolerance of L_T_ and L_S_ was previously confirmed utilizing the identical experimental setup as the present research study, based on survival rate and several growth parameters, such as fresh weight and radicle and coleoptile length [4]. L_T_ displayed the highest level of tolerance among the tested genotypes—there was no statistically significant difference in fresh weight, or radicle and coleoptile length, while the survival rate was 100% in both control and low-temperature conditions. On the other hand, L_S_ had the lowest survival rate (73%) and fresh weight as well as radicle and coleoptile length decreased by ≈35% in the treated seedlings.

After germinating under optimal conditions (25/20 °C (12/12 h), relative humidity (RH) 75%) in the dark in a growth chamber (MLR-352H-PE Climate Chamber, PHC Europe B.V., Breda, The Netherlands), 5-day-old seedlings of both lines were exposed to the following low-temperature conditions for 24 h: 10/8 °C, 12/12 h light/dark photoperiod, light intensity (LI) 700 µmol/m^2^/s. For the analysis of sugar and phenolic compound content, as well as the antioxidative enzyme activity, samples of 30 seedlings per line were taken immediately after 24 h of treatment, frozen, and ground in liquid nitrogen. The samples were stored at −80 °C until further use. Control seedlings were grown under optimal conditions (25/20 °C, 12/12 h photoperiod, LI 700 µmol/m^2^/s, RH 75%) during the same period and sampled at the same time point.

Additionally, a subset of 30 seedlings per line was sown in pots containing a mixture of soil and sand (3:1) and grown in the growth chamber under the previously described optimal conditions for another seven days to recover, after which the photosynthetic efficiency was measured.

### 2.2. Phenolic Compound Content

Total phenolic content (TPC) was determined using the Folin–Ciocalteu (FC) method as described by Waterhouse [22] and Burgos et al. [23]. Briefly, phenolic compounds were extracted through sonication (Branson Ultrasonics, Danbury, CT, USA) in 80% methanol. The results were expressed as gallic acid equivalents per gram of sample (mg GAE/g sample), using the gallic acid standard curve. Flavonoid extraction was performed in the same manner as the total phenolic compound extraction, and total flavonoid content (TFC) determination was conducted by applying methods developed by Meyers et al. [24] and Kostić et al. [25]. The TFC was expressed as quercetin equivalents per gram of sample (mg QE/g sample), utilizing the quercetin standard curve.

The extracts for the detection and quantification of individual phenolic compounds and phenolic acids were prepared according to Pantelić et al. [26], and the compounds of interest were separated and quantified using liquid chromatography–mass spectrometry (LC/MS) and by applying the method described by Radović et al. [27]. The LC-MS/MS analysis utilized a Dionex Ultimate 3000 UHPLC system equipped with a diode array detector (DAD) and a TSQ Quantum Access Max triple-quadrupole mass spectrometer (ThermoFisher Scientific, Basel, Switzerland). The elution was performed on a Syncronis C18 column (100 × 2.1 mm, 1.7 μm) (ThermoFisher Scientific, Basel, Switzerland) at 40 °C. The mobile phases consisted of water with 0.1% formic acid (A) and 100% acetonitrile (B), applied in the following gradient elution: 0–2.0 min—95% A, 5% B; 2.0–14.0 min—95–5% A, 5–95% B; 14.0–14.2 min—5–95%, 95–5% B; 14.2–20.0 min—95% A, 5% B. The mass spectrometer, equipped with a heated electrospray ionization (HESI) source, was used with the following ion source settings: spray voltage 5000 V, sheath gas (N_2_) pressure 40 AU, ion sweep gas pressure 1 AU, auxiliary gas (N_2_) pressure 8 AU, capillary temperature 300 °C, and skimmer offset 0 V. The MS data was acquired in negative mode, in the 100 to 1000 m/z range. Multiple mass spectrometric scanning modes, including full scanning (FS) and product ion scanning (PIS), were applied for the qualitative analysis of the targeted compounds. The collision-induced fragmentation experiments were performed using argon as the collision gas. Instrument control, data acquisition and analysis were carried out using Xcalibur software version 2.2 (Thermo Fisher Scientific, San Jose, CA, USA), while ChemDraw version 12.0 (Revvity Signals Software, Inc., Waltham, NH, USA) was used as a reference library. The total amounts of each compound were evaluated by calculating the peak areas and are expressed as mg/kg.

### 2.3. Soluble Sugar and Sugar Alcohol Compound Content

The contents of individual soluble sugars and sugar alcohols were determined using high-performance anion chromatography with pulsed amperometric detection (HPAEC-PAD), calibrated with standard solutions of various sugars and sugar alcohols: glucose (Glu), fructose (Fru), sucrose (Suc), raffinose (Raf), stachyose (Sta), galactose (Gal), trehalose (Tre), sorbitol (Sor), inositol (Ino), mannitol (Man) and erythritol (Ery) (Tokyo Chemical Industry, TCI, Europe, Antwerp, Belgium). Stock solutions of individual sugars and sugar alcohols (1000 ng/mL) were prepared by dissolving standards in ultrapure water (Millipore Simplicity 185 S.A., Molsheim, France), and then diluted to working solution concentration ranges, which corresponded to the content of each sugar in the samples (0.9–100 ng/mL, depending on the concentration in the samples).

Extraction was performed as per the method described by Waisi et al. [28]. The seedling samples were oven-dried at 130 °C (Carbolite Gero GmbH & Co. KG, Neuhausen, Germany), and 0.25 g was suspended in 5 mL of ultrapure water, ultrasonicated (30 min), and centrifuged (6000× *g*, 10 min). The supernatants were collected and filtered through a 0.45 μm polytetrafluoroethylene membrane filter (Supelco, Bellefonte, PA, USA).

The chromatographic separations were carried out on an ICS 3000 DP liquid chromatography system equipped with a quaternary gradient pump (Dionex, Sunnyvale, CA, USA). The sugar and sugar alcohol compounds were separated at 30 °C, on a Carbo Pac^®^ PA100 pellicular anion-exchange column (4 × 250 mm, 8.5 μm, <10 Å) (Dionex, Sunnyvale, CA, USA). The mobile phases consisted of 600 mM sodium hydroxide (A), 500 mM sodium acetate (B), and ultrapure water (C), applied at a linear gradient (0.7 mL/min) in the following order: 0–5.0 min—15% A, 85% C; 5.0–5.1 min—15% A, 2% B, 83% C; 5.1–12.0 min—15% A, 2% B, 83% C; 12.0–12.1 min—15% A, 4% B, 81% C; 12.1–20.0 min—15% A, 4% B, 81% C; 20.0–20.1 min—20% A, 20% B, 60% C; 20.1–30.0 min—20% A, 20% B, 60% C. The system was pre-conditioned with 15% A, 85% C, for 15 min, and each sample (25 μL) was injected using an ICS AS-DV 50 auto-sampler (Dionex). The electrochemical detector consisted of gold as the working electrode and Ag/AgCl as the reference electrode [28].

### 2.4. Antioxidative Enzyme Activity

Activities of the following antioxidative enzymes were analyzed after the low-temperature treatment: APX, CAT, GPX, POD, and SOD. The antioxidative enzyme activities were calculated based on the previously determined concentration of proteins in the samples, for which the Bradford assay was applied, as well as the calibration curve developed based on known amounts of the standard bovine serum albumin (BSA) solution at 596 nm. Proteins were extracted from 1 g of plant tissue using an extraction buffer (10 mM Tris HCl, pH 8,1; 10 mM EDTA, pH 8.0; 5 mM β-mercaptoethanol; 0.1 mg/mL PMSF).

The activity of APX was calculated based on the concentration of monodehydroascorbate, synthesized from ascorbic acid and H_2_O_2_ in a reaction catalyzed by APX, at 290 nm. Determination of CAT activity was performed through determination of peroxomolybdate concentration, a compound that is synthesized in the presence of CAT and ammonium heptamolybdate, with the absorbance maximum at 405 nm. GPX catalyzes the reaction between reduced glutathione and H_2_O_2_, and the activity of this enzyme was determined by analyzing the reduction in GSH level at 412 nm. POD activity was determined based on the absorbance of pyrogallol quinone, a product of H_2_O_2_ reduction and pyrogallic acid oxidation catalyzed by POD, at 420 nm. SOD activity was measured indirectly by determining the inhibition rate of hydroxylamine oxidation at 550 nm, as SOD inhibits this reaction. All the antioxidative enzyme activity assays were performed using 0.02 g of ground seedling sample, following the manufacturer’s instructions for commercial kits produced by Elabscience^®^ (Wuhan, China): Peroxidase (POD) Activity Assay Kit; Ascorbate Peroxidase (APX) Activity Assay Kit; Glutathione Peroxidase (GPX) Activity Assay; Total Superoxide Dismutase (T-SOD) Activity Assay Kit (Hydroxylamine Method); and Catalase (CAT) Activity Assay Kit. The results of all enzyme activity assays were presented as the amounts of enzyme required to catalyze the reaction with 1 μg of substrate in 1 mg of protein per minute at 37 °C (U/mg_prot_).

### 2.5. Photosynthetic Efficiency

Photosynthetic efficiency after the LT treatment was assessed by measuring the chlorophyll fluorescence parameters after seven days of recovery, using a portable fluorometer Mini-PAM Photosynthesis Yield Analyzer (Walz, Effeltrich, Germany). The analyzed parameters included the photosystem II maximum quantum yield (F_v_/F_m_), photosystem II photochemical efficiency (Φ_PSII_), quantum efficiency of unregulated energy dissipation in PSII (Y(NO)), quantum efficiency of regulated dissipation of energy in PSII (Y(NPQ)), and electron transfer rate (ETR). The values of these parameters were calculated according to Kitajima and Butler [29] and Genty et al. [30].

Chlorophyll fluorescence was measured in treated and control plants after a recovery period of seven days when the maize plants were mostly in the V2 stage of development. Photosynthetic efficiency measurements were performed on each plant using healthy second and third maize leaves, and the measurement for each plant was calculated as the mean of two readings. Measurement of F_v_/F_m_ was carried out on leaves adapted to conditions of total darkness for 30 min, while all the other parameters were measured in full light conditions. Total darkness conditions were ensured using specific Mini-PAM attachments (Dark Leaf Clip DLC-8, Walz, Effeltrich, Germany). After adaptation to the dark, the minimum fluorescent signal (F_0_) as well as the maximum fluorescence signal (F_M_) were measured, and these parameters were used to calculate the maximum quantum yield of photosystem II, using the formula: F_v_/F_m_ = (F_M_ − F_0_)/F_M_. For light-adapted plants, stable light-adapted fluorescence (F) and maximum light-adapted fluorescence (F_M_’) were obtained with a special Leaf Clip Holder 2035-B accessory (Walz, Effeltrich, Germany). These values were used for calculating Φ_PSII_, Y(NO), Y(NPQ), and ETR using the following formulas: Φ_PSII_ = (F_M_’ − F)/F_M_’; Y(NO) = F/F_M_’; Y(NPQ) = Y(NO) − F/F_M_; and ETR = Φ_PSII_ · PAR · α · β (where α is the fraction of incident photons absorbed by the panel of the fluorometer (α = 0,84); β is the relative distribution of absorbed PAR to photosystem II (β = 0.5); PAR is the quantum flux density of photosynthetically active radiation impinging on the sample). The data was collected and processed using the software provided with the device, WinControl-3 (version 3.23, Walz, Effeltrich, Germany).

### 2.6. Statistical Analysis

All experimental analyses were performed in triplicates. The data were recorded as mean ± standard deviation and analyzed in R (v4.3.3) [31]. The statistical significance of the difference between the control and treatment conditions was calculated by performing Student’s *t*-test with a significance level at *p* < 0.05. Log normalization (log(x + 1)) was applied to the results, and principal component analysis (PCA) was performed to calculate the distances and determine the relationships between individual samples using factoextra (v1.0.7) [32] in R. To compute trait–trait correlations, Spearman correlations were calculated using the R function Hmisc (v5.2.3) [33], with a cutoff of correlation coefficient *r* = 0.7 and FDR-adjusted *p* = 0.05 for significance. Significant correlations were visualized using Cytoscape software (v3.10.2) [34]. Additionally, after calculating z-scores from the log-normalized results, hierarchical clustering was performed using the cluster R package (v2.1.6) [35] and a heatmap was constructed using the R-function ComplexHeatmap (v2.18.0) [36,37].

## 3. Results

### 3.1. Phenolic Compound Content

**Figure 1:**
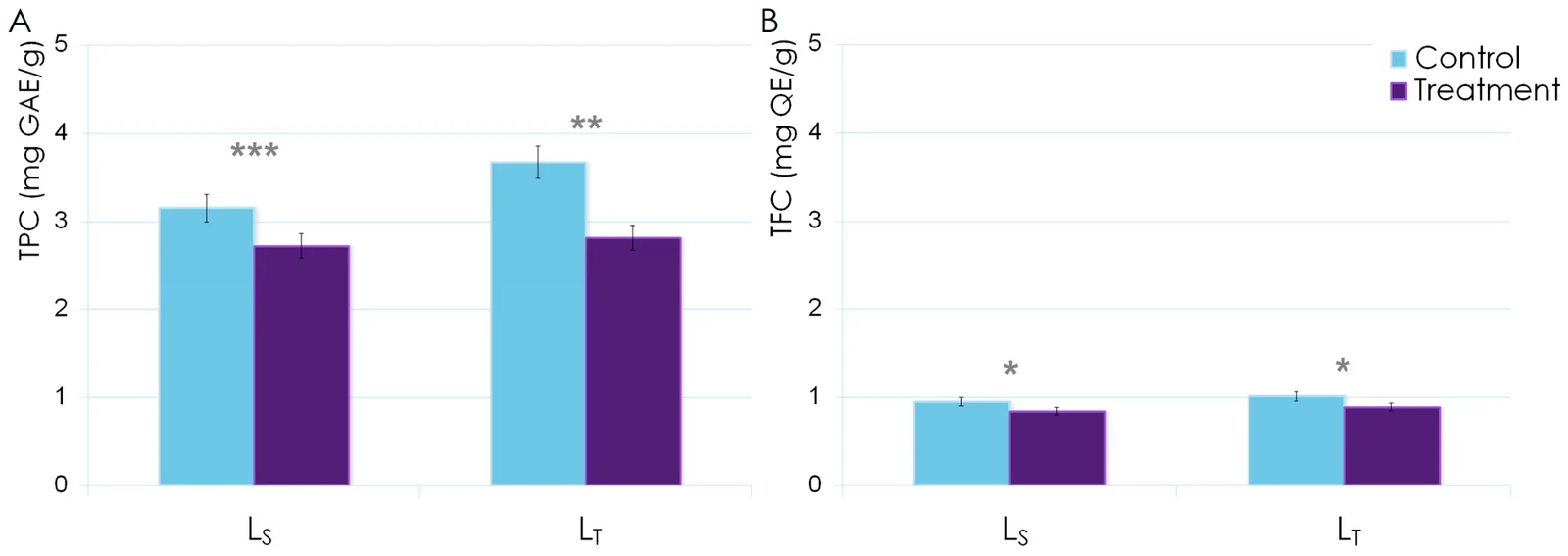
Changes in total phenolic and flavonoid contents caused by low-temperature stress. (**A**) Total phenolic content (TPC) in control and treatment conditions in the tolerant (L_T_) and susceptible (L_S_) genotypes. (**B**) Total flavonoid content (TFC) in control and treatment conditions in L_T_ and L_S_ genotypes. TPC and TFC in control and treatment samples are indicated by teal and pink, respectively. The standard deviation is shown as an error bar at the top of each column in the graph. Statistical significance of the difference between control and treatment was determined by Student’s *t*-test and is shown as *** (*p* < 0.001), ** (*p* < 0.01), * (*p* < 0.05).

The TPC decrease in the treated samples was statistically significant in both genotypes (Figure 1A). TPC was 3.15 mg GAE/g in the control plants of inbred line L_S_, while in the treated plants, it was 2.72 mg GAE/g—a decrease of ≈15%. In L_T_, TPC was ≈24% lower in the treated samples (L_T_ C 3.69 mg GAE/g, L_T_ T 2.82 mg GAE/g). Similar statistically significant results were also observed for the flavonoid content—TFC was reduced by ≈12% in the treated plants of both genotypes (L_S_ C 0.95 mg QE/g, L_S_ T 0.84 mg QE/g, L_T_ C 1.01 mg QE/g, L_T_ T 0.89 mg QE/g) (Figure 1B).

**Figure 2:**
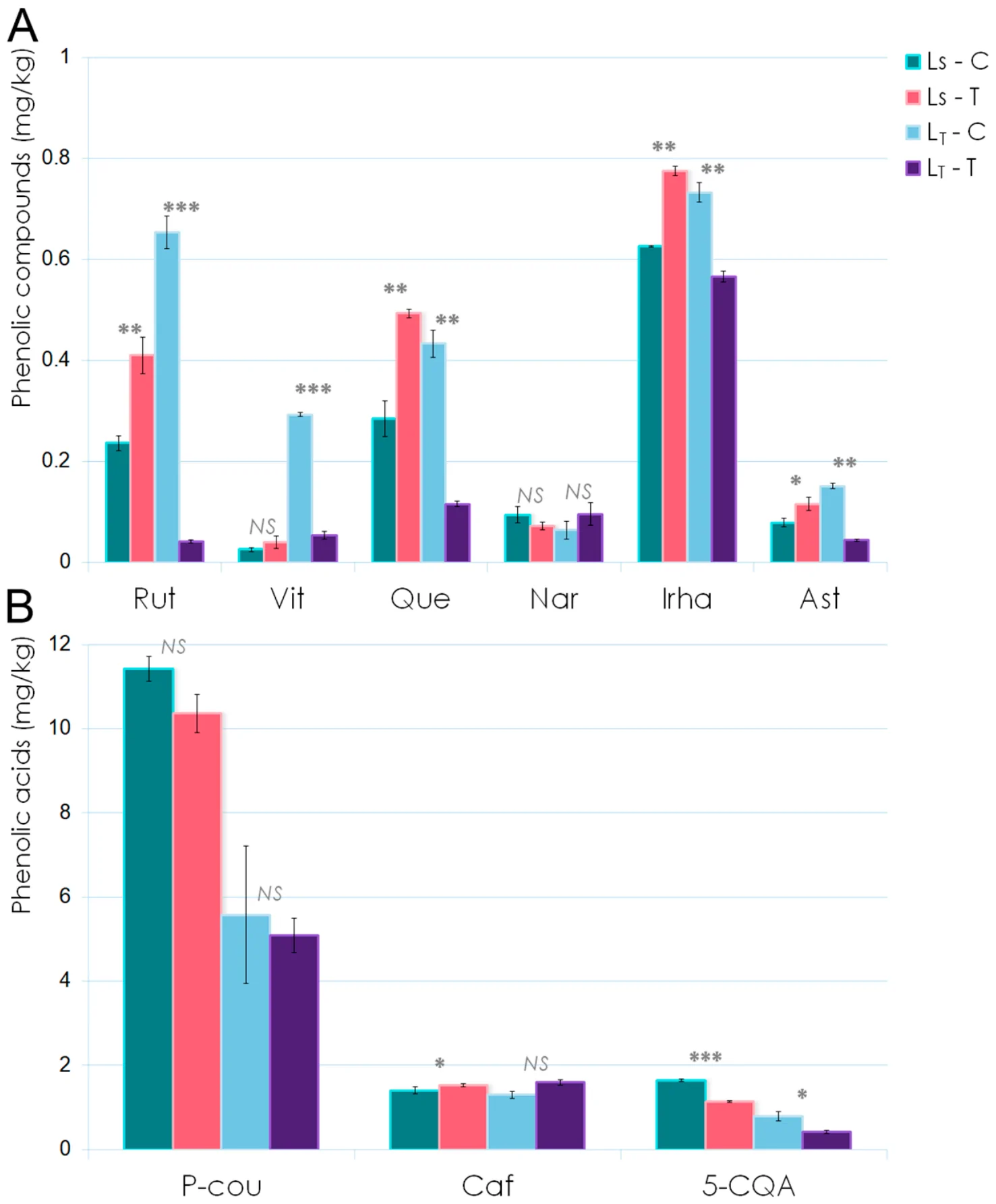
Changes in the contents of phenolic compounds and acids caused by low-temperature stress. (**A**) Phenolic compound content. The detected phenolic compounds were rutin (Rut), vitexin (Vit), quercetin (Que), naringenin (Nar), isorhamnetin (Irha), and astragalin (Ast). (**B**) Phenolic acid content. The detected phenolic acids were p-coumaric acid (P-cou), caffeic acid (Caf), and neochlorogenic acid (5-CQA). The contents in the control samples of the susceptible genotype (L_S_) are shown in teal (L_S_–C); treatment samples of L_S_ in pink (L_S_–T); control samples of the tolerant genotype (L_T_) in blue (L_T_–C); and treatment samples of L_T_ in purple (L_T_–T). The standard deviation is shown as an error bar at the top of each column in the graph. Statistical significance of the difference between control and treatment was determined by Student’s *t*-test and is shown as *** (*p* < 0.001), ** (*p* < 0.01), * (*p* < 0.05) and NS (*p* > 0.05).

Individual phenolic compounds detected in the samples were rutin (Rut), vitexin (Vit), quercetin (Que), naringenin (Nar), isorhamnetin (Irha), and astragalin (Ast). There was no statistically significant difference between the control and treatment conditions in either of the genotypes in the naringenin content, while vitexin content significantly differed between the conditions only in L_T_ (*p* < 0.001). The contents of rutin, vitexin, quercetin, isorhamnetin, and astragalin were all increased in the treated samples of L_S_ compared to the control ones: a ≈70% increase in the rutin and quercetin levels, ≈20% in the isorhamnetin level, and ≈40% in the level of astragalin. An opposite trend was observed in the L_T_ genotype: the levels of rutin (≈93%), quercetin (≈75%), vitexin (≈80%), isorhamnetin (≈20%) and astragalin (≈67%) were all significantly lower in the treated samples. The content of the listed phenolic compounds in both lines is shown in Figure 2A.

Additionally, p-coumaric (P-cou), caffeic (Caf), and neochlorogenic acid (5-CQA) were also found in the maize seedlings during emergence. No statistically significant difference in P-cou content was found between the control and treatment conditions in either genotype. Caffeic acid content was significantly increased only in the treated samples of L_S_ (≈10%), while the level of 5-CQA was lower in treated plants of both inbred lines (L_S_ ≈ 30%, L_T_ ≈ 50%). The content of the phenolic acids in both lines is shown in Figure 2B.

### 3.2. Soluble Sugar and Sugar Alcohol Content

Several of the analyzed soluble sugars showed similar accumulation profiles in both lines when comparing the control and treatment conditions. Specifically, significantly higher contents of fructose, sucrose, and raffinose were observed in the control samples of both genotypes. Fructose levels were lowered by ≈30% in the treated seedlings of both maize lines. The changes in sucrose levels were more pronounced—sucrose levels were three and five times lower in the treated seedlings of genotypes L_T_ and L_S_, respectively. On the other hand, while the level of raffinose decreased by only ≈30% in the treated seedlings of L_T_, it was two times lower in the treated seedlings of L_S_. Additionally, both stachyose and trehalose followed the same accumulation profiles in both lines, but in this case, the content was higher in the treated samples. In L_S_, the levels of both stachyose and trehalose were higher in treated plants by ≈40% and ≈20%, respectively. This increase was statistically significant for both sugars. Changes observed in the L_T_ line were less pronounced—≈10% in the level of both sugars. However, the difference between control and treatment was statistically significant only for trehalose.

**Figure 3:**
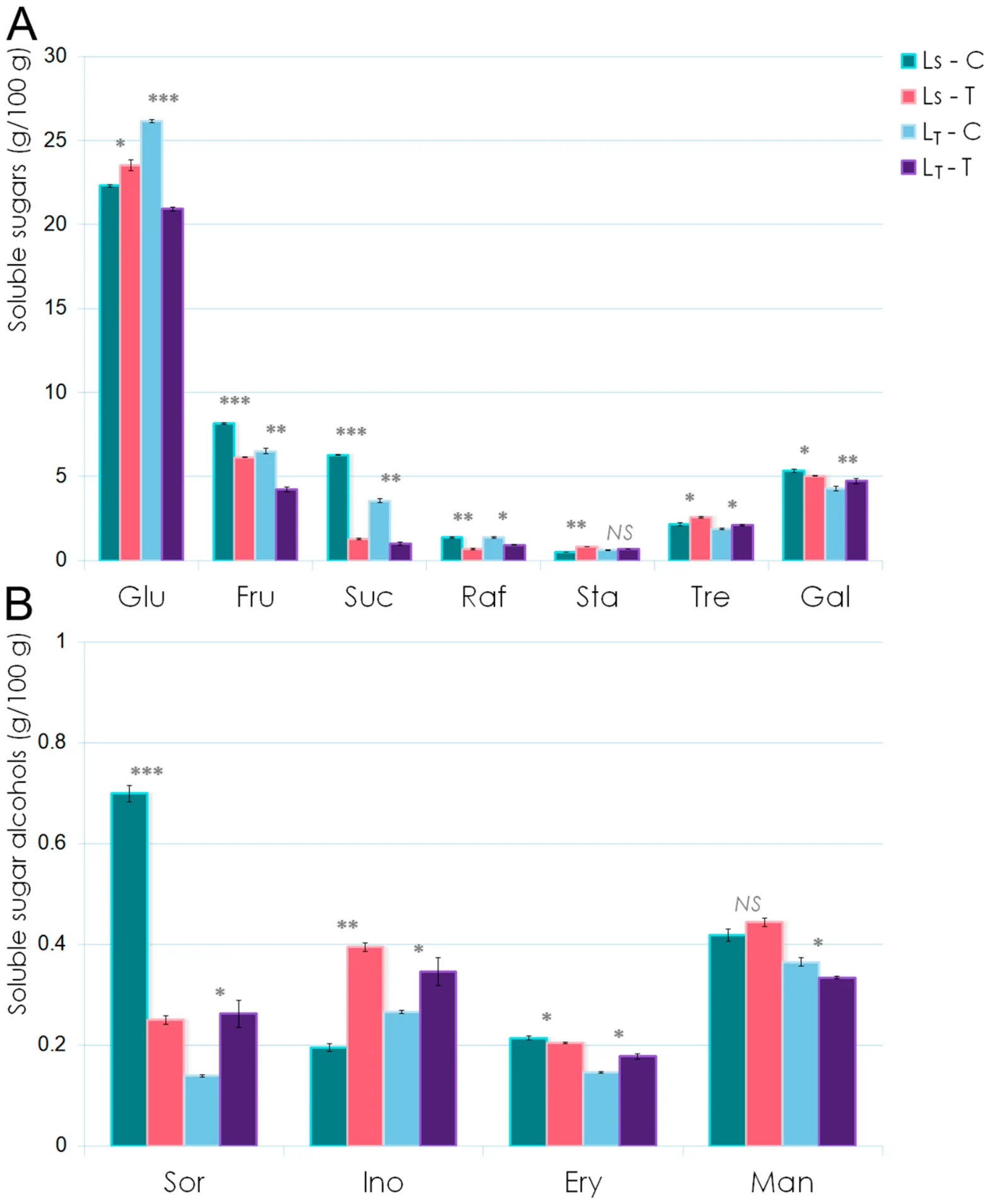
Changes in the soluble sugar and sugar alcohol contents caused by low-temperature stress. (**A**) Soluble sugar content. The analyzed soluble sugars were glucose (Glu), fructose (Fru), sucrose (Suc), raffinose (Raf), stachyose (Sta), trehalose (Tre), and galactose (Gal). (**B**) Soluble sugar alcohol content. The analyzed soluble sugar alcohols were sorbitol (Sor), inositol (Ino), erythritol (Ery), and mannitol (Man). The contents in the control samples of the susceptible genotype (L_S_) are shown in teal (L_S_–C); treatment samples of L_S_ in pink (L_S_–T); control samples of the tolerant genotype (L_T_) in blue (L_T_–C); and treatment samples of L_T_ in purple (L_T_–T). The standard deviation is shown as an error bar at the top of each column in the graph. Statistical significance of the difference between control and treatment was determined by Student’s *t*-test and is shown as *** (*p* < 0.001), ** (*p* < 0.01), * (*p* < 0.05) and NS (*p* > 0.05).

The remaining two sugars, glucose and galactose, showed opposite accumulation patterns in the two inbred lines. Glucose content increased (≈5%) in the treated plants of L_S_ and control plants of L_T_ (≈20%). On the contrary, galactose content increased (≈7%) in the treated samples of L_T_ and control samples (≈10%) of L_S_. All the differences between the control and the treatment were statistically significant. The content of the analyzed soluble sugars in both lines is shown in Figure 3A.

The analyzed soluble sugar alcohols and their contents are shown in Figure 3B. Sugar alcohols were present in smaller amounts than sugars, but the differences between control and treated samples were more pronounced. Apart from inositol, which was significantly increased in both L_T_ (≈30%) and L_S_ (≈100%), the genotypes showed opposite patterns in the accumulation of sugar alcohols. A statistically significant increase in the treated plants was found in sorbitol (≈100%) and erythritol (≈20%) in L_T_, while they were significantly reduced in the treated plants in L_S_—erythritol by ≈5% and sorbitol by ≈70%. On the other hand, mannitol changes under treatment were statistically significant only in L_T_, where it was reduced by ≈10%, while a statistically insignificant increase of ≈5% was detected in L_S_.

### 3.3. Antioxidative Enzyme Activity

**Figure 4:**
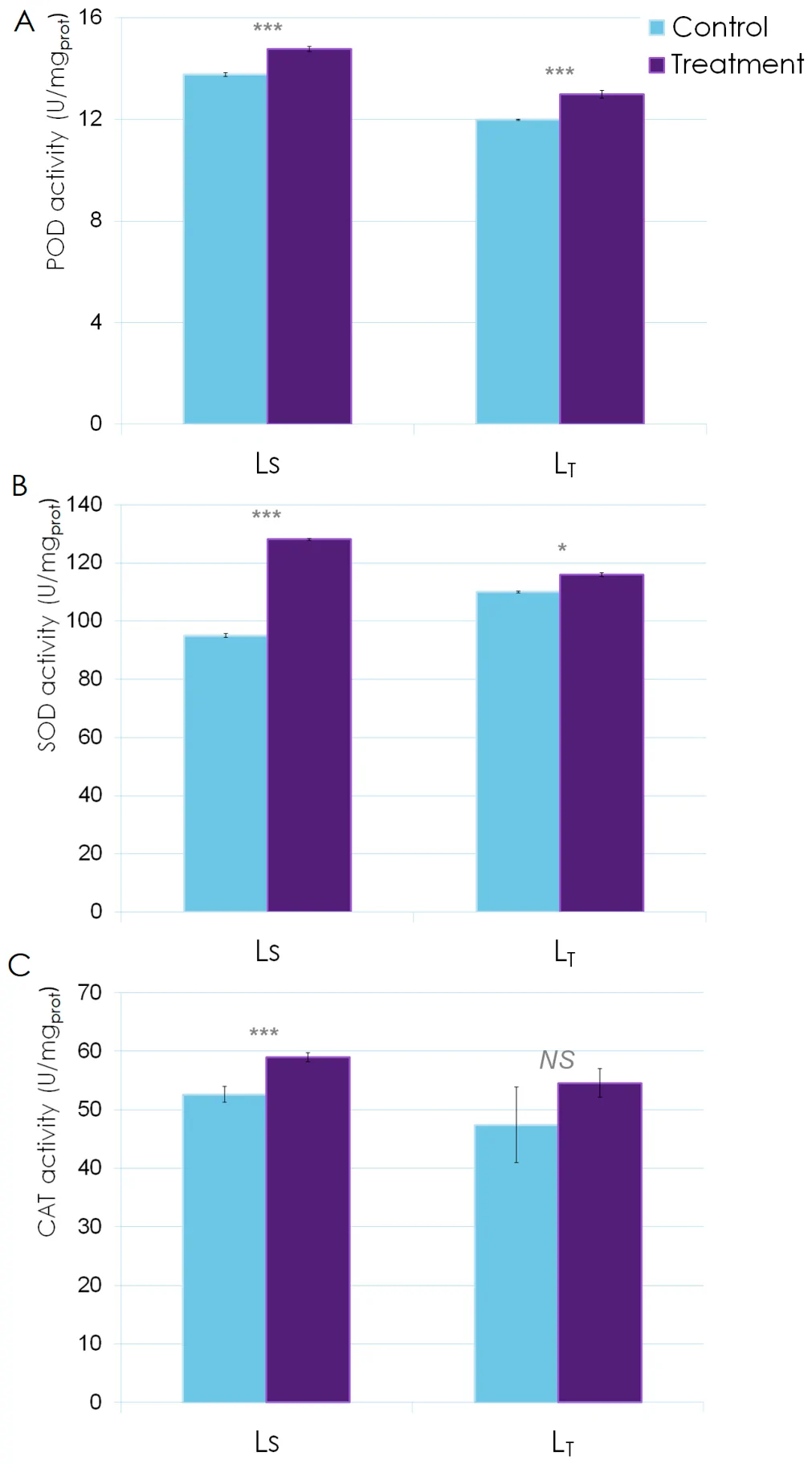
Changes in the peroxidase (POD), superoxide dismutase (SOD) and catalase (CAT) activity caused by low-temperature stress in the tolerant (L_T_) and susceptible (L_S_) genotypes. (**A**) POD activity. (**B**) SOD activity. (**C**) CAT activity. Enzyme activities in control and treatment samples are represented by teal and pink bars, respectively. The standard deviation is shown as an error bar at the top of each column in the graph. Statistical significance of the difference between control and treatment was determined by Student’s *t*-test and is shown as *** (*p* < 0.001), * (*p* < 0.05) and NS (*p* > 0.05).

The activity of most of the analyzed antioxidative enzymes (POD, SOD, CAT) was higher in the treated samples of both genotypes (Figure 4). There was a statistically significant, but weakly pronounced increase in the level of POD activity in the samples treated with low temperatures. The POD activity increased by less than 10% in the treated samples of both genotypes. In L_S_, the activity rose from 13.76 to 14.77 U/mg_prot_, while in L_T_, it increased from 11.98 to 12.98 U/mg_prot_. The SOD activity increase in the treated samples was statistically significant in both lines, but it was less pronounced in L_T_. While a 25% increase was detected in L_S_ (from 95 to 128.1 U/mg_prot_ in control and treated samples, respectively), only a 5% increase occurred in L_T_ (from 110.1 to 115.9 U/_t_ in control and treated samples, respectively). When it comes to CAT activity, the increase in the treated samples was more pronounced in the tolerant (≈15%) than in the susceptible (≈10%) genotype, although the change was statistically significant only in L_S_. The measured activities increased from 47.4 to 54.6 U/mg_prot_ in L_T_, and from 52.6 to 59 U/mg_prot_ in L_S_.

**Figure 5:**
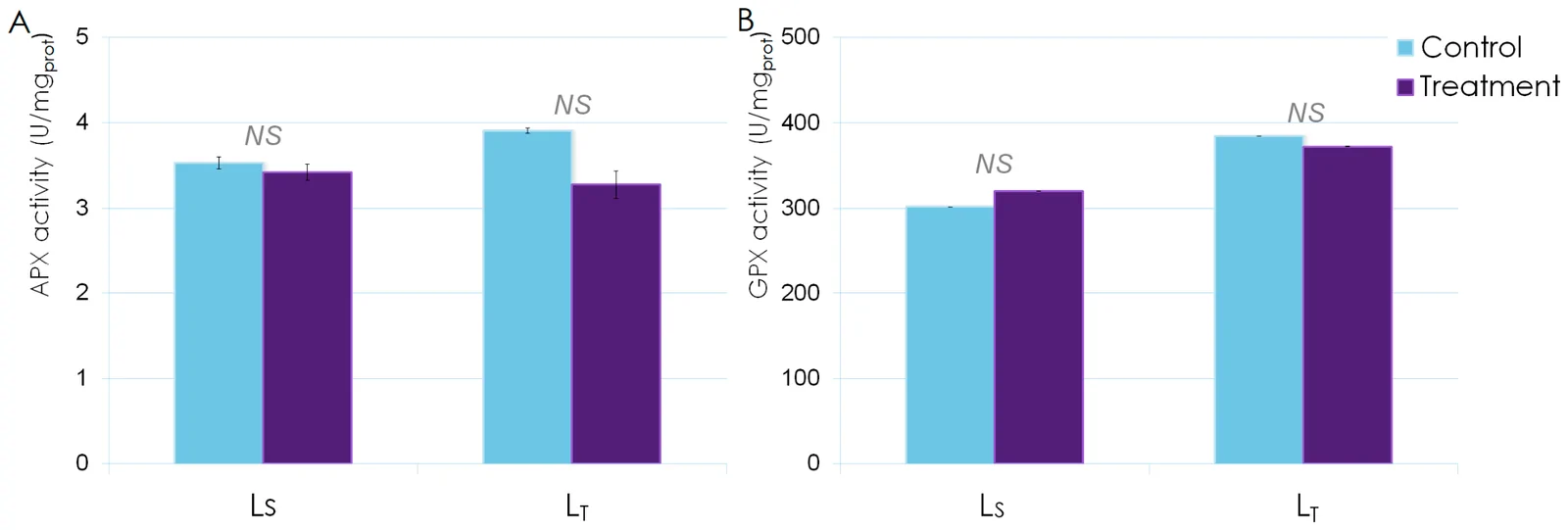
Ascorbate peroxidase (APX) and glutathione peroxidase (GPX) activity caused by low-temperature stress in the tolerant (L_T_) and susceptible genotype (L_S_). (**A**) Ascorbate peroxidase (APX) activity. (**B**) Glutathione peroxidase (GPX) activity. The antioxidative enzyme activity is expressed in units of activity per milligram of protein (U/mg_prot_). Enzyme activities in control and treatment samples are represented by teal and pink bars, respectively. The standard deviation is shown as an error bar at the top of each column in the graph. Statistical significance of the difference between control and treatment was determined by Student’s *t*-test and is shown NS (*p* > 0.05).

There was no statistically significant difference in the activity of APX and GPX between the control and treatment samples in either of the genotypes (Figure 5). A decrease in the activity of APX was found in treated seedlings of both genotypes. In L_S_, the activity decreased by less than 5%—from 3.53 in control to 3.42 U/mg_prot_ in treatment conditions. In L_T_, the decrease in APX activity was more pronounced (≈20%)—from 3.91 in control to 3.28 U/mg_prot_ in treated seedlings. GPX activity showed different patterns in L_S_ and L_T_ under the treatment. A decrease of less than 5% was found in treated plants of L_T_ (from 384.4 to 372.4 U/mg_prot_), while an increase of 6.1% in GPX activity was detected in L_S_ (from 301.7 to 320.1 U/mg_prot_).

### 3.4. Photosynthetic Efficiency

In order to determine the photosynthetic efficiency, F_v_/F_m_, Φ_PSII_, Y(NO), Y(NPQ) and ETR were calculated, and the results are shown in Table 1. Statistically significant differences were observed only in the F_v_/F_m_ and Φ_PSII_ in L_S_, with the values of both parameters reduced in treated plants (≈5%).

### 3.5. Principal Component, Clustering and Correlation Analysis

**Figure 6:**
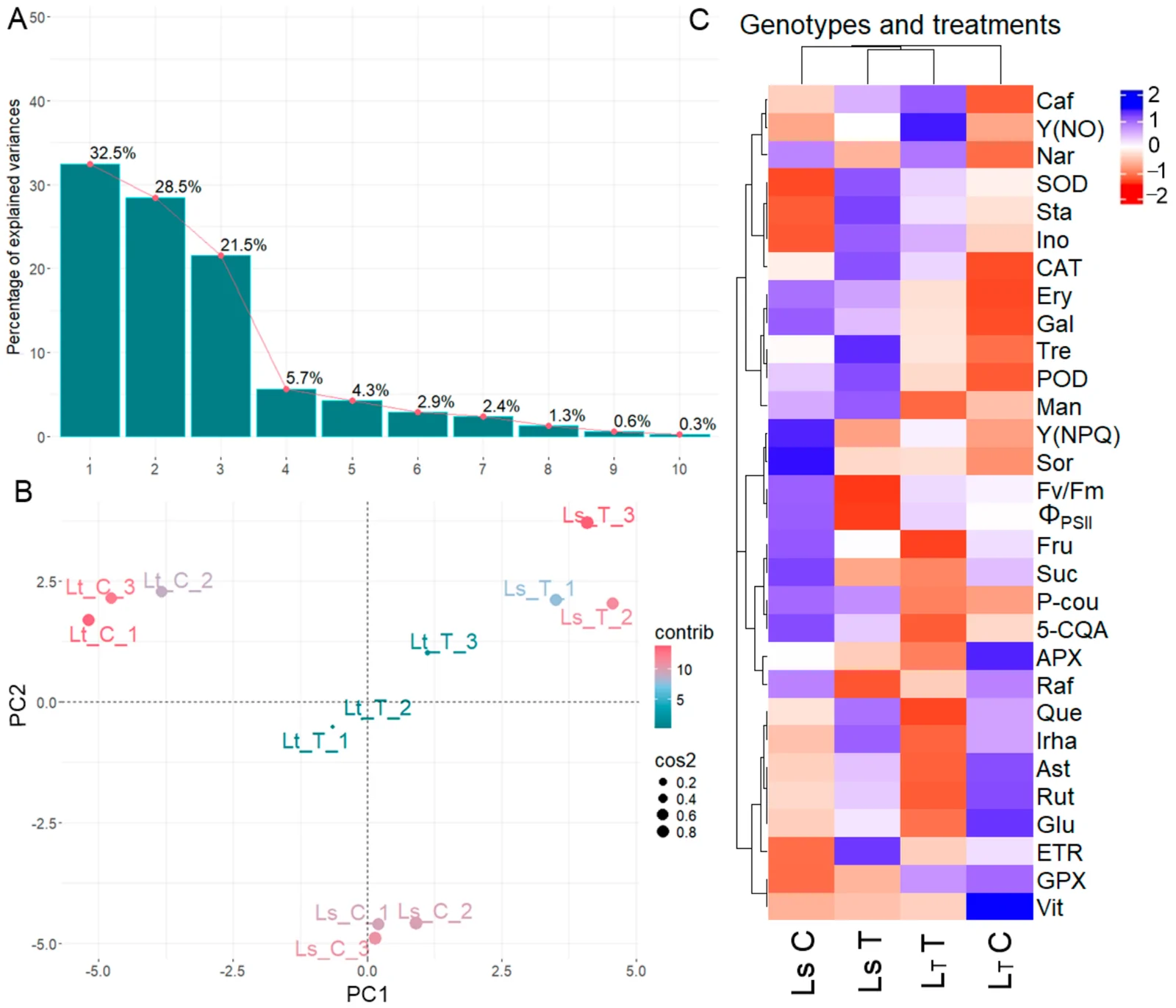
Principal component and clustering analysis. (**A**) Percentage of explained variances of selected principal components (PCs). (**B**) Principal component analysis (PCA) plot of individual samples, explained by the first two PCs (PC1 and PC2). The contributions of the variables are shown in the range 0 (teal)—15 (pink), while the cos2 values of the variables are shown as different point sizes ranging from 0.2 to 0.8. Individual measurements in the triplicates are labeled as 1, 2, and 3. (**C**) Hierarchical cluster analysis based on z-scores calculated from the log(x + 1) values of the analyzed traits across the four samples. Z-scores ranged from −2 (red) to 2 (blue).

Principal component analysis revealed that the first three principal components accounted for more than 80% of the total variability of the analyzed traits (Figure 6A). Principal component 1 (PC1) accounted for 32.5% of the explained variance, and the traits that contributed the most to PC1 were 5-CQA, Tre, POD, Vit, and Ery. On the other hand, principal component 2 (PC2) explained 28.5% of the total variability, to which Sor, SOD, Sta, Ino, and Φ_PSII_ contributed the most.

**Figure 7:**
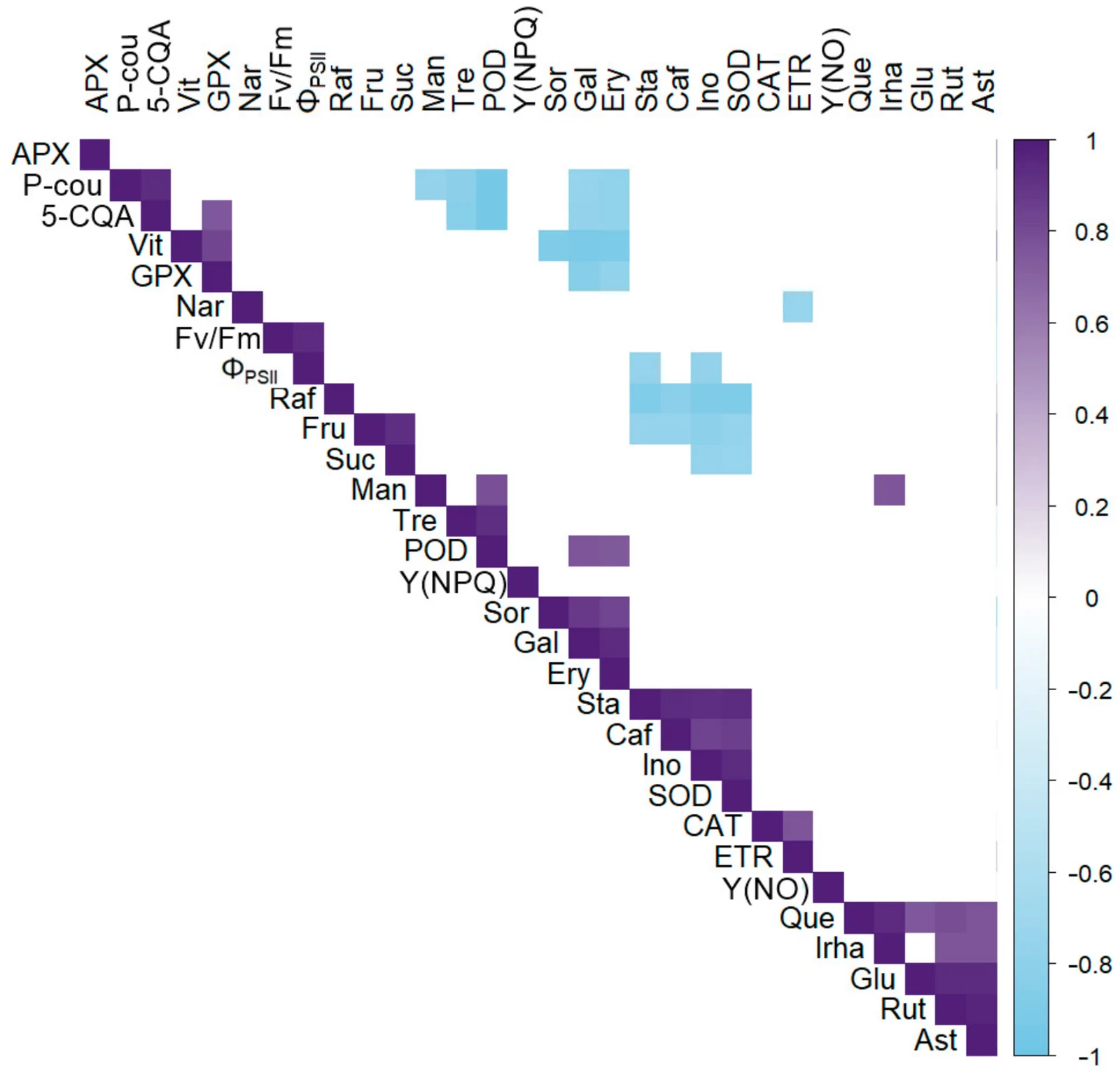
Spearman’s correlation coefficient matrix of traits involved in the low-temperature response. The correlation matrix shows only significant (adjusted *p*-value < 0.05) and strong (*r* > 0.7) correlations for flavonoid, phenolic acid, sugar, and sugar alcohol profiles, as well as antioxidative enzyme activities and photosynthetic efficiency in four analyzed samples (L_S_ C, L_S_ T, L_T_ C, and L_T_ T). The correlation strength and direction are shown on the bar on the right. Positive correlations are shown in teal, negative in pink, while the correlation strengths are reflected in the color opacity.

**Figure 8:**
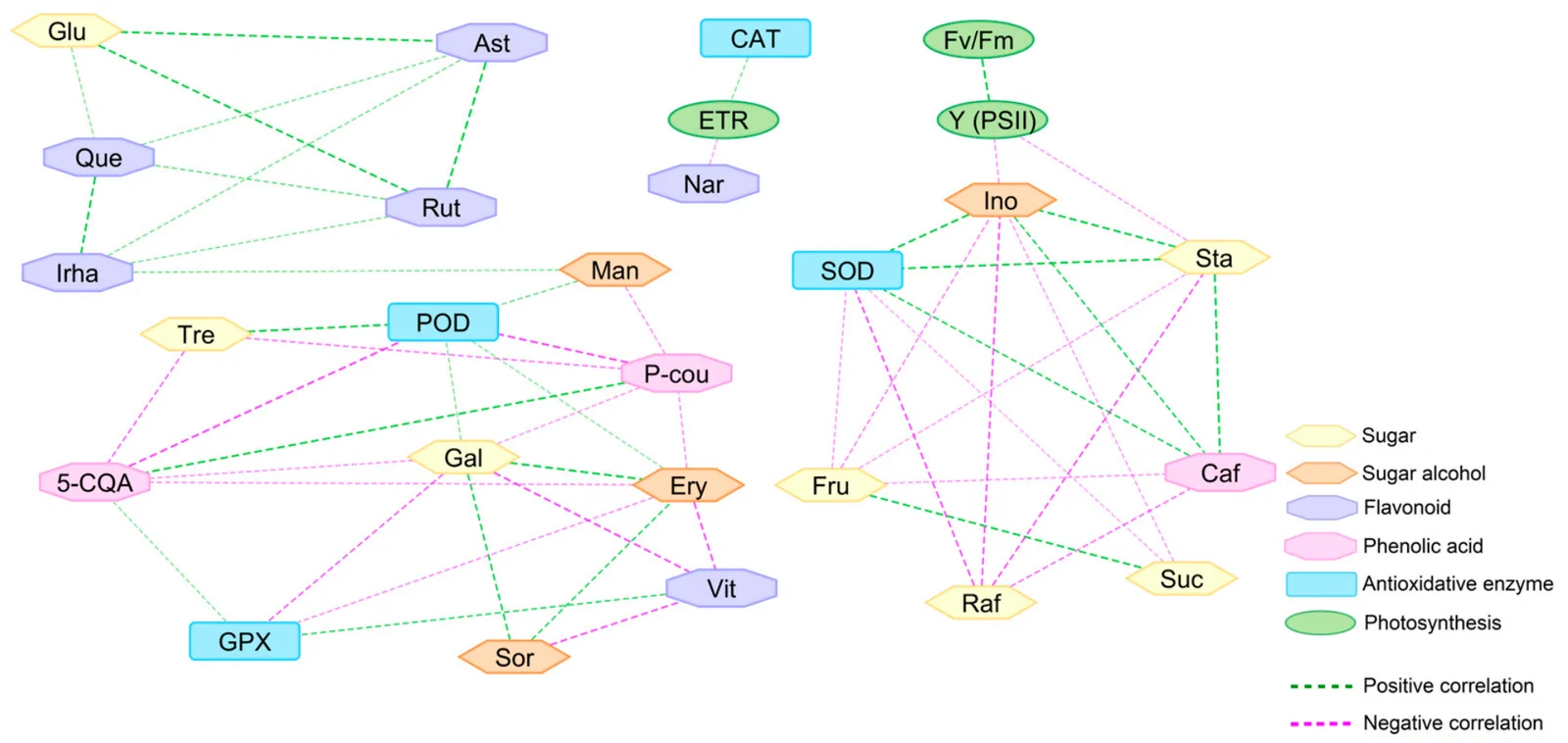
Interaction network of the traits involved in the low-temperature response based on Spearman’s correlation coefficients. Sugars are presented as yellow hexagons; sugar alcohols as orange ones; octagons represent flavonoids (purple) and phenolic compounds (pink); while antioxidative enzymes are shown as blue rectangles and photosynthetic efficiency parameters as green ellipsoids. Positive correlations are shown in green dashed lines, while negative correlations are shown in magenta. The adjusted *p*-values of the correlations are presented as the transparency levels of the dashed lines—lower adjusted *p*-values equate to less transparent lines, and vice versa.

The PCA plot (Figure 6B) shows a clear distinction among the genotypes and treatment conditions based on PC1 and PC2. The tolerant and susceptible genotypes were clearly separated in the control conditions based on both PC1 and PC2, and this was further confirmed by the cluster analysis (Figure 6C). However, in the treatment conditions, the two genotypes clustered together. Spearman’s correlation coefficient (*r*) matrix is shown in Figure 7 and depicts only the significant correlations among many of the analyzed traits in the four samples. The significant correlations are further visualized in the form of a network in Figure 8. All the Spearman’s correlation coefficients and the adjusted *p*-values are shown in Table S1, available as Supplementary Materials.

Glu showed a strong positive correlation with certain flavonoids—Que (*r* = 0.76), Ast (*r* = 0.94), and Rut (*r* = 0.93). Additionally, Irha accumulation was positively correlated with Que (*r* = 0.94), Rut (*r* = 0.76) and Ast (*r* = 0.77). POD activity was positively correlated with the accumulation of Tre (*r* = 0.92) and Gal (*r* = 0.76), which also exhibited a positive correlation with Sor (*r* = 0.87) and Ery (*r* = 0.94). SOD activity was also positively correlated with certain metabolites—Ino (*r* = 0.77), Caf (*r* = 0.77) and Sta (*r* = 0.77)—while all of them were in negative correlation with Fru and Raf.

## 4. Discussion

### 4.1. Phenolic Compound Content

Both TPC and TFC significantly decreased following LT treatment in both maize genotypes, despite phenolic compounds generally being associated with enhanced tolerance to abiotic stress [18,38]. Phenolic compounds in general are accumulated under stress conditions in various plant species, including maize [39], and flavonoids in particular are considered key components of the LT stress response [19,40]. Similar reductions in phenolic compounds under abiotic stress have also been reported previously. The total flavonoid content in basil plants decreased under LT treatment, while the total phenolic content increased during the first 24 h and was then reduced below the control level [41]. A decrease in the level of these secondary metabolites was also recorded under other types of abiotic stress—oilseed rape under drought [42] and maize exposed to high soil salinity [43]. However, TPC and TFC alone are insufficient indicators of stress tolerance, as the methods applied for their detection could impact the results. The method of tissue preparation and the isolation method itself, as well as the type of tissue and the life stage, influence the obtained values [44]. Therefore, it is necessary to examine the influence of low temperatures on the accumulation of individual phenolic metabolites in more detail.

Although total phenolic and flavonoid contents declined in both genotypes, the profiles of individual phenolic compounds differed markedly between the tolerant and susceptible lines, suggesting genotype-specific regulation of phenylpropanoid metabolism. Rutin, quercetin, isorhamnetin and astragalin accumulated only in the treated seedlings of L_S_, and their accumulation profiles were positively correlated, which was not surprising as they are all products of naringenin in the phenylpropanoid pathway [45]. The opposite, although not statistically significant, was found in L_T_, with only the naringenin level increasing under LT conditions. Additionally, the genes encoding the enzyme responsible for transforming naringenin into dihydroflavonols (flavanone 3-dioxygenase) or kaempferol into quercetin (flavonoid 3’-monooxygenase) were downregulated [5]. Data on the rutin and quercetin accumulation in response to LT stress are limited. Previous studies have reported inconsistent effects of LT on rutin accumulation, with increases observed in tomato [44] and buckwheat [46], whereas Suzuki et al. [47] reported no significant changes. These contrasting findings suggest that flavonoid responses are highly species- and context-dependent. For example, flavonols and their glucosides are known to influence the auxin gradient and level in cells, and thus the development of plant organs [48]. Quercetin is a known auxin inhibitor [49] and can also modulate root growth by limiting cell proliferation [50]. Thus, flavonoids and their derivatives may play a role in the repression of auxin-induced activities, i.e., growth and morphogenesis, and stimulation of stress response mechanisms. In our previous research [4], it was shown that low temperatures had a significant negative impact on fresh weight, as well as radicle and coleoptile length in L_S_. Therefore, the increased rutin and quercetin coincided with reduced seedling growth under LT, suggesting that these flavonoids may be linked to growth inhibition through modulation of auxin signaling. On the other hand, there was no statistically significant difference in fresh weight and radicle and coleoptile length between the control and treated plants of the tolerant genotype, and no accumulation of these flavonoids in the treated plants. The results suggest that LT tolerance at this stage may depend more on maintaining growth than on the accumulation of the phenolic metabolites analyzed in this study. However, the confirmation of such results requires additional analyses.

Like all phenolic compounds, phenolic acids play a role in the response to various abiotic stress factors due to their antioxidant properties, but during emergence under LT treatment, the changes in phenolic acid content were not significantly pronounced. For example, caffeic acid content was significantly increased only in treated seedlings of L_S_. Additionally, a gene encoding caffeic acid 3-O-methyltransferase, responsible for the transformation of caffeic acid to ferulic acid, was downregulated in L_S_ [5]. Although increased caffeic acid accumulation is often associated with tolerance to various stress factors in a number of plant species, including low temperatures [51,52], there is not much data for maize, as most studies have focused on drought response. For example, drought can lead to a decrease [53] or an increase in the level of caffeic acid [54]. Additionally, caffeic acid is known to increase SOD activity under other abiotic stress factors and positively impact ROS elimination [52]. In the present study, it was shown that there was a positive correlation between caffeic acid content and SOD activity. Furthermore, SOD activity was more pronounced in the treated seedlings of L_S_, pointing towards the possible role of caffeic acid in improving the enzymatic antioxidative response under LT conditions during emergence. However, this may reflect a compensatory response to greater oxidative stress rather than an indicator of enhanced tolerance, and confirming either of the possibilities requires further research.

### 4.2. Soluble Sugar Content

The accumulation profiles of soluble sugars in this study were generally the same regardless of the genotype, except for glucose and galactose. While soluble sugars are known to be important osmoprotectants that help maintain the activity of biological macromolecules and participate in ROS removal and cell membrane stabilization under low-temperature conditions [14], in this research, it was necessary to also take into account the life stage in which the tissue was sampled. Maize seedlings at this stage are still predominantly heterotrophic, meaning growth and development largely depend on the availability of sugars in seed reserves [55,56]. Still, the photosynthetic apparatus is also being established, and photosynthesis is occurring at a low rate. Consequently, sugars present in plant cells at this stage predominantly originate from seed reserves, while photosynthetically produced sugars gradually accumulate during development. Research on such early developmental stages is not common, and there is not much data on soluble sugar levels during abiotic stress. The available data point to total soluble sugar content decreasing in LT conditions during emergence [57,58]. The results of transcriptomic profiling were consistent with this, as many genes encoding enzymes of the Calvin cycle (*pep1*, *prk*, *rca1*, *fbp2*), gluconeogenesis (*fbp2*, *fba*), and sugar transport (*sweet*) were downregulated [4,5]. In the present study, when it comes to individual sugars, a similar pattern was observed for fructose, sucrose and raffinose in both L_S_ and L_T_, which suggests that sugar metabolism during emergence may differ from that of fully developed plants.

The susceptible genotype accumulated more glucose following the LT treatment, while the opposite was found in the tolerant genotype. Glucose is a specific soluble sugar, as it acts not only as an osmoprotectant, but as a signaling molecule as well. It is known that glucose can interact with different phytohormones and modulate processes essential for growth and development, as well as responses to changes in the environment [59,60]. Glucose can inhibit auxin-mediated processes, such as root formation and cell elongation, as well as seed germination and early development [61,62]. Transcriptomic profiling has also revealed that several components of the auxin pathway were inhibited in L_S_, including an auxin efflux carrier (*pin5b*) and an auxin-responsive protein (*SAUR52*) [4,5]. Also, glucose is significantly interconnected with the phenylpropanoid pathway, acting primarily as the ultimate carbon source for phenylpropanoid synthesis and is necessary for rutin biosynthesis [47]. In the case of L_S_, the increased glucose level and positive correlation with flavonoids that can negatively affect growth and development (rutin, quercetin, astragalin) could also be associated with the reduced growth parameters measured in the treated seedlings of the L_S_ genotype [4].

Galactose accumulation profiles also differed between the genotypes, increasing only in L_T_. Additionally, zma-miR168b-3p, which targeted two putative UDP-glucose 4-epimerases, was upregulated in both genotypes, but the upregulation was much more pronounced in L_S_, suggesting a potential significance in maintaining a specific balance between UDP-galactose and UDP-glucose under LT conditions [4]. There is limited information on galactose accumulation under abiotic stress conditions. The galactose level decreased in poplar under high salinity conditions [63], and in cucumber plants exposed to low temperatures [64]. Also, in maize, galactose possibly participates in drought response as a component of galactinol, which is necessary for the synthesis of raffinose [65]. The present study suggests that it may also contribute in establishing LT tolerance as well, but more research is needed to confirm such findings.

Raffinose level declined in treated seedlings, but its product, stachyose, accumulated in both the tolerant and susceptible genotypes under LT conditions, although it was significant only in L_S_. Low-temperature treatment is known to induce the synthesis of both raffinose and stachyose, and accumulation of raffinose under LT conditions is associated with tolerant crop varieties [64,66,67,68]. As raffinose is synthesized from sucrose, a possible explanation for the raffinose decrease under LT treatment could be the lack of sufficient amounts of its precursor, as sucrose accumulation is also lowered in the treated plants of both genotypes. Also, a negative correlation was found between raffinose and stachyose, suggesting that stachyose accumulation could potentially be contributing to the raffinose decrease as well. Another possible explanation is that sucrose needed for raffinose synthesis was preferentially allocated to other metabolic processes, such as primary cell wall development, although this needs to be further investigated.

A significant finding was the increase in trehalose content in treated seedlings of both genotypes, as well as a positive correlation between this increase and POD activity. Among the analyzed sugars, trehalose is best known for its role in the adaptation and response of plants to various environmental changes, especially low temperatures. It plays a significant role in cellular integrity protection, stabilization of dehydrated enzymes and membranes, and antioxidative response enhancement [69,70,71]. Specifically for maize, it has been shown that low-temperature treatment induces trehalose synthesis and accumulation [72]. These findings further support previous reports and suggest that trehalose plays an important role in cellular signaling during LT response, regardless of the genotype’s tolerance to this stress factor.

### 4.3. Soluble Sugar Alcohol Content

Genotypes L_S_ and L_T_ differed significantly in soluble sugar alcohol content under low-temperature treatment. Three of the four analyzed sugar alcohols were detected in higher amounts in the treated seedlings of the tolerant genotype (sorbitol, inositol, and erythritol), but not in L_S_.

Mannitol was the only sugar alcohol that accumulated in L_S_, but not in L_T_ seedlings, after LT treatment. In contrast, mannitol content decreased significantly in the treated seedlings of L_T_. The role of mannitol in establishing tolerance to various stress factors has been demonstrated primarily through seed or plant pretreatment experiments [73,74,75]. However, the data on the role of mannitol in LT response is limited. Previous research showed that there was no significant increase in mannitol content during acclimation to LT [76], but that pretreatment with mannitol improved antioxidant capacities in LT-treated plants [77]. All the sugar alcohols analyzed in the present study are synthesized from fructose-6-phosphate [78], so it is possible that the different synthesis pathways were prioritized in the two genotypes, affecting their overall LT response. In L_T_, the carbon flux may have been directed towards synthesizing sorbitol, erythritol and inositol under LT treatment, possibly limiting the availability of Fru-6-P for mannitol synthesis, but more research is necessary to confirm that.

Sorbitol and inositol are known to play important roles in osmotic adjustment under abiotic stress across a large number of plant species [79,80], so their accumulation in L_T_ following low-temperature treatment was not surprising. Additionally, low temperatures are known to induce both sorbitol [81,82] and inositol synthesis [83,84,85]. Sorbitol accumulation patterns in L_S_ and L_T_ could be connected with glucose profiles. Specifically, one of the pathways of sorbitol synthesis includes the reduction of glucose or its product, glucose-6-phosphate [80,86]. These results suggest that glucose may have been converted to sorbitol only in L_T_ seedlings, while this pathway may be less active in L_S_, leading to a poorer LT response. Also, in L_T_ the genes encoding sorbitol transporters were downregulated [5], possibly to ensure high intercellular levels of this sugar alcohol, while in L_S_, the miRNA zma-miR164e-5p that targets putative polyol transporters was upregulated [4]. Additionally, inositols are precursors of inositol phosphates that are essential parts of the inositol–phospholipid signaling pathway, necessary for an effective abiotic stress response [79]. The fact that these compounds accumulated in both the tolerant and susceptible genotypes suggests that they play an important role in LT response during emergence, but this needs to be further researched.

Erythritol content increased only in the tolerant genotype following treatment, but erythritol is a less common sugar alcohol and its role in plant responses to environmental changes remains poorly understood. Accumulation of erythritol has been recorded under abiotic stress in several species of grasses tolerant to desiccation [87,88], but reports on maize are scarce. Erythritol was detected in very small amounts in several maize cultivars in a limited number of studies [89,90]. The higher erythritol content observed only in treated LT seedlings, and the positive correlation between erythritol accumulation and galactose and sorbitol levels, may suggest a potential contribution to the low-temperature response during emergence. However, confirming this would require additional analyses of the regulatory mechanism behind erythritol accumulation under abiotic stress, which is additionally complicated due to the incomplete characterization of biosynthetic pathways in maize.

### 4.4. Antioxidative Enzyme Activity

Low-temperature treatment induced an antioxidative response in both genotypes. POD, SOD, and CAT activities increased significantly in L_S_ following treatment, whereas only POD and SOD activities were increased in L_T_. Peroxidases contribute to the maintenance of H_2_O_2_ homeostasis [91], and low temperatures are known to increase their activity in maize [92,93,94]. Superoxide dismutases complement the activity of peroxidases, as they are responsible for the disproportionation of superoxide radicals [95]. SOD activity is also increased under low-temperature conditions in maize [9,12]. The increased POD and SOD activities observed in the treated seedlings of both genotypes were consistent with previous research, which could also indicate their significance in the low-temperature response during emergence. On the other hand, the increase in CAT activity following LT treatment was statistically significant only in the susceptible genotype. Catalases, along with POD, are the key enzymes responsible for the catalytic neutralization of H_2_O_2_, with CAT usually being more active at higher concentrations of this molecule [95]. CAT activity increases in response to various abiotic stress factors, and this has been confirmed in various studies of abiotic stress response in maize, including low temperatures [58,94]. The stronger CAT response in L_S_ may reflect the previously noted fact that this enzyme is more active at higher concentrations of H_2_O_2_. It is possible that the treatment temperatures did not lead to oxidative stress of the same intensity in the L_T_ as in the L_S_ genotype, resulting in CAT activity not being increased to the same extent as in the susceptible genotype.

In contrast to POD, SOD and CAT, no statistically significant differences in APX and GPX activities were observed between the control and treatment in either genotype. Like POD and CAT, APX has a role in neutralizing H_2_O_2_ [96], so it is possible that antioxidative capacities of POD and CAT were sufficient for the LT response during emergence. Additionally, Ramazan et al. [93] determined that APX in maize has specific time patterns of accumulation—it is most active in the first 8 h of exposure to low temperatures. Therefore, it is also possible that APX was involved in the initial stress response, after which APX activity decreased, but further research is necessary to confirm this. Glutathione peroxidases are widely distributed enzymes that play a role in the detoxification of lipid peroxides and other ROS [97], which has been observed in various plant species under low-temperature conditions [98,99]. However, the information regarding the role of GPX in response to abiotic stress factors in maize remains limited. GPX activity has been studied under increased cadmium concentrations [100] and salt levels [101], but not low temperatures. The lack of a significant difference between GPX activity in control and treatment conditions in both analyzed genotypes suggests that GPX may play a limited role in antioxidative response during emergence in maize, although its involvement at other stages cannot be excluded.

### 4.5. Photosynthetic Efficiency

Seven days following the low-temperature treatment, there was no significant difference between control and treatment for most parameters (Y(NO), Y(NPQ), ETR). However, a statistically significant difference was observed for F_v_/F_m_ and Φ_PSII_ only in L_S_—the values of these parameters were lower in the treated seedlings.

It is well known that low-temperature stress significantly inhibits photosynthesis, particularly in C4 plants with a more sensitive photosynthetic apparatus, such as maize [102]. In particular, the initial light-dependent reactions occurring within PSI and PSII are very sensitive to low temperatures [103]. Low temperatures reduce the number of functional PSII reaction centers, thereby limiting the maximum photon density that can be used for energy transformation and reducing the electron transfer capacity. Additionally, under moderate to high irradiance, lipid peroxidation of chloroplast membranes and chlorosis occur, resulting in photoinhibition [104]. Chlorophyll fluorescence parameters (F_v_/F_m_, Φ_PSII_, Y(NO), Y(NPQ), ETR) are widely used indicators of PSII efficiency, with F_v_/F_m_ serving as the most informative measure of PSII photochemical efficiency [15]. Numerous research studies have confirmed the negative influence of low temperatures on the photosynthetic electron transport efficiency in maize using these parameters [105,106]. However, a reduction in photosynthetic efficiency does not necessarily indicate irreversible photodamage. Under some conditions, it reflects a protective downregulation of PSII, provided that excess excitation energy is efficiently dissipated as heat through non-photochemical quenching (Y(NPQ)) [15]. Given that Y(NPQ) did not increase in L_S_ following treatment, the observed reductions in F_v_/F_m_ and Φ_PSII_ are unlikely to represent a protective measure, and instead suggest photoinhibition induced by low temperatures. No significant changes in chlorophyll fluorescence parameters were observed in plants of genotype L_T_, suggesting either reduced damage to the photosynthetic apparatus, or a more efficient recovery process. This interpretation is supported by transcriptome profiling of maize seedlings during emergence, which revealed a stronger downregulation of genes involved in the establishment and proper functioning of PSI and PSII in the susceptible genotype compared to L_T_. While many of the *psa*, *psb*, *lhc*, and *cab* genes were downregulated in both genotypes, the number was much higher in L_S_—nine in L_T_, and 18 in L_S_. Additionally, several genes encoding proteins involved in the regulation of the Calvin cycle and Rubisco activity, as well as carbon fixation, were downregulated, potentially further contributing to the disruption of the light-dependent stages of photosynthesis, like the gene encoding Rubisco activase (*rca*) or phosphoribulokinase (*prk*). Additionally, the *pep1* gene encoding a phosphoenolpyruvate carboxylase, a low-temperature-sensitive enzyme characteristic of C4 metabolism, was downregulated in both genotypes, but the change was more pronounced in L_S_. The transcriptome profiling also revealed that L_T_ seemed to be more efficient in handling photoinhibition, with significant upregulation of genes encoding proteins and enzymes with protective roles, such as *ctpa2*, *elips*, *grxs*, and *uf3gt*, limiting the damage caused by LT [5].

## 5. Conclusions

The analysis of biochemical and physiological parameters in seedlings during emergence following exposure to low temperatures provided insights into the differences in the responses between the tolerant and susceptible maize varieties. The results showed that the treatment temperatures did not represent a stress of equal intensity for both genotypes. The extent of the negative impact of low temperatures on the normal progression of metabolic processes was observed through the significant decrease in photosynthetic efficiency only in L_S_ (F_v_/F_m_ and Φ_PSII_). Additionally, in the treated seedlings of the susceptible genotype, there were increased levels of molecules with known functions in growth inhibition and activation of signaling pathways responsible for stress response—rutin, quercetin, isorhamnetin, astragalin, and glucose. Such patterns were not observed in L_T_.

Also, the metabolites deployed to limit the damage caused by low temperatures are determined by the developmental stage at which the low-temperature stress occurs. During emergence, the synthesis of non-enzymatic antioxidants (polyphenolic compounds) and osmoprotectants (sugars and sugar alcohols) is based on the transformation of already available reserve substances. This could explain the decrease in the levels of total phenolic and flavonoid compounds, as well as of several of the analyzed sugars (fructose, sucrose, raffinose), in the treated L_S_ and L_T_ seedlings. Nevertheless, certain defense mechanisms were common to both genotypes—accumulation of trehalose and inositol, increased activity of POD and SOD. Certain differences in response between the genotypes were also observed. In the tolerant genotype, sugar alcohols (sorbitol, erythritol, inositol) seemed to have a bigger role in osmoprotection and were accumulated in the treated seedlings, in contrast to L_S_. On the other hand, in the susceptible genotype, antioxidative enzyme activities (POD, SOD, CAT) were more pronounced compared to L_T_, which could be connected to the impaired photosynthetic efficiency, detected only in the susceptible genotype.

This research suggests that during emergence, securing fast growth and establishment of photosynthesis were important aspects of the low-temperature response. The tolerant genotype seemed to be more efficient at this, and consequently did not seem to require significant accumulation of antioxidants and osmoprotectants, while the same could not be said for the susceptible genotype.

## Figures and Tables

**Table 1:** Difference in photosynthetic efficiency between control and treatment in susceptible (L_S_) and tolerant genotype (L_T_).

Parameter	L_S_			L_T_		
	Control	Treatment	Statistical Significance	Control	Treatment	Statistical Significance
F_v_/F_m_	0.637	0.607	***	0.625	0.627	NS
Φ_PSII_	0.643	0.605	*	0.626	0.631	NS
ETR	45.76	50.81	NS	48.49	47.21	NS
Y(NO)	0.224	0.225	NS	0.224	0.227	NS
Y(NPQ)	0.009	0.004	NS	0.004	0.006	NS

Photosynthetic activity parameters are listed in the first column: maximum quantum yield of photosystem II (F_v_/F_m_), photochemical efficiency of photosystem II (Φ_PSII_), quantum efficiency of unregulated energy release in PSII (Y(NO)), quantum efficiency of regulated energy release in PSII (Y(NPQ)) and electron transfer rate (ETR). Statistical significance of the difference between control and treatment was determined by Student’s *t*-test and is shown as *** (*p* < 0.001), * (*p* < 0.05) and NS (*p* > 0.05).

## Data Availability

The data in this article are available upon request with the consent of the authors.

## Supplementary Materials

The following supporting information can be downloaded at https://www.mdpi.com/article/10.3390/biology15181634/s1: Table S1: Spearman’s correlation coefficients and the adjusted *p*-values for flavonoid, phenolic acid, sugar, and sugar alcohol profiles, as well as antioxidative enzyme activities and photosynthetic efficiency in four analyzed samples (L_S_ C, L_S_ T, L_T_ C, and L_T_ T).

## Abbreviations

The following abbreviations are used in this manuscript:

LT	Low-temperature
L_S_	Susceptible genotype
L_T_	Tolerant genotype
C	Control
T	Treatment
APX	Ascorbate peroxidase
CAT	Catalase
GPX	Glutathione peroxidase
POD	Peroxidase
SOD	Superoxide dismutase
PSII	Photosystem II
F_V_/F_m_	Photosystem II maximum quantum yield
ETR	Electron transfer rate
ROS	Reactive oxygen species
RH	Relative humidity
LI	Light intensity
TPC	Total phenolic content
FC	Folin–Ciocalteu
TFC	Total flavonoid content
LC/MS	Liquid chromatography–mass spectrometry
DAD	Diode array detector
HESI	Heated electrospray ionization
FS	Full scanning
PIS	Product ion scanning
HPAEC-PAD	High-performance anion chromatography with pulsed amperometric detection
Glu	Glucose
Suc	Sucrose
Fru	Fructose
Raff	Raffinose
Sta	Stachyose
Gal	Galactose
Tre	Trehalose
Ino	Inositol
Man	Mannitol
Sor	Sorbitol
Ery	Erythritol
BSA	Bovine serum albumin
Φ_PSII_	Photosystem II photochemical efficiency
Y(NO)	Quantum efficiency of unregulated energy dissipation in photosystem II
Y(NPQ)	Quantum efficiency of regulated dissipation of energy in photosystem II
F_0_	Minimum fluorescent signal
F_M_	Maximum fluorescent signal
F_M_’	Maximum light-adapted fluorescent signal
PCA	Principal component analysis
Rut	Rutin
Vit	Vitexin
Que	Quercetin
Nar	Naringenin
Irha	Isorhamnetin
Ast	Astragalin
P-cou	P-coumaric acid
Caf	Caffeic acid
5-CQA	Neochlorogenic acid
PC	Principal component
*r*	Spearman’s correlation coefficient
